# New recommendations for rhythm control—What has changed in the 2023 ACC/AHA/ACCP/HRS and 2024 ESC guidelines for atrial fibrillation, and where does dronedarone fit in?

**DOI:** 10.1016/j.ahjo.2025.100645

**Published:** 2025-10-20

**Authors:** Gerald V. Naccarelli, Justin Rackley, Giuseppe Boriani

**Affiliations:** a Heart and Vascular Institute, Pennsylvania State University College of Medicine, Hershey, PA, USA; b Cardiology Division, Department of Biomedical, Metabolic and Neural Sciences, University of Modena and Reggio Emilia, Modena, Italy

**Keywords:** AF guidelines, Antiarrhythmic drugs, Atrial fibrillation, Dronedarone, Rhythm control

## Abstract

In recent years, the importance of early rhythm control to delay the progression of atrial fibrillation (AF) has been recognized, as have the benefits of catheter ablation and antiarrhythmic drugs (AADs) as first-line therapy for rhythm control. Selecting the most appropriate AAD according to its safety profile as well as individual patient characteristics is of key importance. To inform decision-making, up-to-date guidelines are paramount. The American College of Cardiology (ACC)/American Heart Association (AHA)/American College of Clinical Pharmacy (ACCP)/Heart Rhythm Society (HRS) guidelines for AF were updated in 2023, while the European Society of Cardiology (ESC) guidelines for AF were updated in 2024.

Dronedarone is an AAD indicated in the US to reduce the risk of hospitalization for AF in patients in sinus rhythm with a history of paroxysmal or persistent AF. In Europe, it is indicated for the maintenance of sinus rhythm after successful cardioversion in clinically stable adults with paroxysmal or persistent AF. Since the last major review of the efficacy and safety of dronedarone (published in 2019), multiple real-world evidence (RWE) studies and post hoc analyses of key dronedarone randomized controlled trials have been performed. This review discusses the findings of these RWE studies and post hoc analyses in the context of the 2023 ACC/AHA/ACCP/HRS and 2024 ESC guidelines for AF with a focus on dronedarone as a treatment option for early rhythm control, its use after catheter ablation, and its use in people with heart failure and a mildly reduced or preserved ejection fraction.

## Abbreviations

**Table:** 

AAD	Antiarrhythmic drug
ACC	American College of Cardiology
ACCP	American College of Clinical Pharmacy
AF	Atrial fibrillation
AFL	Atrial flutter
AHA	American Heart Association
ATA	Atrial tachyarrhythmia
CA	Catheter ablation
CHA_2_DS_2_-VASc score	Congestive HF, hypertension, age, diabetes, prior stroke, transient ischemic attack, or thromboembolism, vascular disease, age, sex category
CKD-EPI	Chronic Kidney Disease Epidemiology Collaboration
CV	Cardiovascular
CYP3A4	Cytochrome P450 enzyme 3A4
ECG	Echocardiogram
ESC	European Society of Cardiology
GDMT	Guideline-directed medical therapy
HCRU	Healthcare resource utilization
HF	Heart failure
HFmrEF	Heart failure with mildly reduced ejection fraction
HFpEF	Heart failure with preserved ejection fraction
HFrEF	Heart failure with reduced ejection fraction
HRS	Heart Rhythm Society
LVEF	Left ventricular ejection fraction
MI	Myocardial infarction
NOAC	Non–vitamin K antagonist oral anticoagulant
NPR	National Patient Register
NYHA	New York Heart Association
pHR	Pooled HR
PSM	Propensity score matched
SR	Sinus rhythm
VA EHR	US Veterans Health Administration electronic health records

## 1 Introduction

Atrial fibrillation (AF) is the most common sustained arrhythmia, with the prevalence in the US expected to reach 12.1 million by 2030 [1]. This is due in part to longer life expectancy [2], improved survival of people with cardiovascular (CV) disease [3], and improved detection of AF [4]. AF is a multifaceted condition requiring management of associated risk factors as well as treatment of the condition itself [5,6]. For many patients with AF, antiarrhythmic drug (AAD) therapy is warranted; however, selecting the most appropriate AAD based on its safety profile as well as individual patient characteristics is of key importance [5,6]. Understanding the most current guidelines on the treatment of AF is essential to inform decision-making.

The American College of Cardiology (ACC)/American Heart Association (AHA)/American College of Clinical Pharmacy (ACCP)/Heart Rhythm Society (HRS) guidelines for AF were updated in 2023 [5]. This was the first full update of the US guidelines since 2014 [7]. In addition, the European Society of Cardiology (ESC) guidelines for AF were updated in 2024 [6]. Both the 2023 ACC/AHA/ACCP/HRS and 2024 ESC guidelines base recommendations for the use of AAD therapy on safety, underlying heart disease, and comorbidities [5,6]. A description of the different classes of recommendations for each guideline is included in the Supplemental Methods.

Key updates in the 2023 ACC/AHA/ACCP/HRS guidelines include the recognition of AF as a progressive disease and an emphasis on the benefits of early rhythm control to prevent disease progression and reduce AF burden [5]. Catheter ablation (CA) is recognized as superior to AAD therapy as a rhythm control strategy in selected patients (ie, those who are younger and who have fewer comorbidities), and patients at earlier stages of heart failure with reduced ejection fraction (HFrEF). As such, the use of first-line CA for rhythm control has been upgraded from a class 2a to a class 1 recommendation for these patients. However, CA and AAD therapy are not mutually exclusive. They are used effectively in combination. The use of AAD therapy to maintain sinus rhythm (SR) or decrease AF burden has been downgraded from a class 1 to a class 2a recommendation, with the choice of AAD being dependent on underlying heart disease and other comorbidities. While CA has been upgraded to a class 1 recommendation for specific patients, AAD therapy may still be preferred over CA, such as when the patient prefers a noninvasive approach or when patient characteristics make CA unfavorable. Additionally, short-term (3–6 months) use of AAD therapy following ablation can reduce early recurrence of atrial arrhythmia and hospitalization, with the 2023 ACC/AHA/ACCP/HRS guidelines giving this approach a class 2a recommendation. In terms of updates to the use of specific antiarrhythmics, the use of sotalol has been downgraded to a class 2b recommendation due to its association with increased risk of mortality; there has not been a change in the recommendation status of other antiarrhythmics.

The 2024 ESC guidelines [6] also have several key updates to the previous (2020) guidelines [8]. The use of early rhythm control therapy (within 12 months of diagnosis) for selected patients with AF at risk of thromboembolic events has been given a class IIa recommendation. In patients with paroxysmal AF who require rhythm control therapy, a class I recommendation is given to both CA and AAD therapy (with drug choice based on presence of HF and comorbidities). Although the class I recommendation for the use of AAD therapy is retained for patients with persistent AF, the use of CA has a class IIb recommendation. However, the choice of which approach to use for rhythm control should ultimately be based on shared decision-making with the patient [6]. In the 2024 ESC guidelines, the use of AAD therapy is not recommended in patients with advanced conduction disturbances unless antibradycardia pacing is provided (class III) [6].

Dronedarone is an AAD that was approved for clinical use in 2009 in both the US and Europe. In the US, dronedarone is indicated to reduce the risk of hospitalization for AF in patients in SR with a history of paroxysmal or persistent AF [9]. In Europe, it is indicated for the maintenance of SR after successful cardioversion in clinically stable adults with paroxysmal or persistent AF [10]. The 2023 ACC/AHA/ACCP/HRS guidelines have assigned dronedarone a class 2a recommendation for the reduction of AF burden in patients without HF, and in those with prior MI, significant heart disease including HFrEF (left ventricular ejection fraction [LVEF] ≤40%) in the absence of NYHA class III/IV HF, or HF with recent decompensation [5]. In the 2024 ESC guidelines, dronedarone has a class Ia recommendation for long-term rhythm control, including in those with heart failure with mildly reduced ejection fraction (HFmrEF), heart failure with preserved ejection fraction (HFpEF), ischemic heart disease, or valvular disease to prevent recurrence and progression of AF [6]. The efficacy and safety of dronedarone were last extensively reviewed in 2019 [11]; however, multiple real-world evidence (RWE) studies and post hoc analyses of key dronedarone randomized controlled trials (RCTs) have since been performed.

To better inform healthcare professionals on the appropriate use of dronedarone for rhythm control, it is important that these data are discussed in detail, and in the context of the 2023 ACC/AHA/ACCP/HRS and 2024 ESC guidelines for AF. This editorial discusses the findings of these newer studies with a focus on dronedarone as a treatment option for early rhythm control, both as an alternative AAD to sotalol (due to similarities in drug indications and because of the downgraded guideline recommendations for sotalol) and for use after CA. We also discuss its use in specific patient populations, namely those with HFmrEF, HFpEF, or NYHA class I/II HF and no recent decompensation.

## 2 Rationale for early rhythm control

### 2.1 AF is now recognized as a progressive disease

AF progression has been associated with increased rates of CV hospitalization and a tendency toward more CV adverse events (AEs) [12]. The 2023 ACC/AHA/ACCP/HRS guidelines have redefined the classification of AF to better recognize it as a progressive disease. The classification describes 4 stages in the AF continuum: at risk for AF, pre-AF, AF, and permanent AF; each stage requires a different management approach [5]. An observational study using the Canadian Registry of Atrial Fibrillation revealed that 10 years after presenting with paroxysmal AF, >50 % of patients had either progressed to persistent AF or died [13]. Based on disease pathology, early treatment of AF to achieve rate and/or rhythm control is key to prevent progression, irreversible atrial cardiomyopathy, and adverse outcomes [5].

### 2.2 Rhythm control therapy is now considered a preferable alternative or adjunct to rate control therapy

Recent studies have recognized that rate and rhythm control should not necessarily be mutually exclusive treatment options and have demonstrated the benefits of early rhythm control for AF [[14], [15], [16], [17], [18]]. EAST-AFNET 4 was an international, investigator-initiated, parallel-group, open, blinded-outcome-assessment study in patients with early AF (diagnosed ≤12 months before enrollment) [17]. It enrolled patients (mean age, 70.3 years) with a previous transient ischemic attack or stroke and ≥ 2 other risk factors for AF [17]. Patients were randomized to receive either early rhythm control (AAD therapy or ablation, as well as cardioversion of persistent AF, initiated early after randomization) or usual care (initial treatment with rate control without rhythm control) [17]. The first co-primary outcome was a composite of death from CV causes, stroke (ischemic or hemorrhagic), or hospitalization with worsening of HF or acute coronary syndrome, analyzed in a time-to-event analysis. The second co-primary outcome was the number of nights spent in hospital per year [17]. At baseline, the primary strategy for rhythm control was AAD therapy, which was used in 86.8 % of patients (35.9 % flecainide, 19.6 % amiodarone, 16.7 % dronedarone, 7.0 % propafenone, and 7.6 % other AAD); CA was used in 8.0 % of patients, and 5.2 % of the cohort did not receive rhythm control [17]. At 2 years after randomization, 45.7 % of patients were receiving AAD therapy (21.0 % flecainide, 11.8 % amiodarone, 5.9 % dronedarone, 3.8 % propafenone, and 3.2 % other AAD), 19.4 % were receiving CA, and 34.9 % were not receiving rhythm control. Early initiation of rhythm control therapy was associated with a 25 % reduction in CV events compared with usual care without affecting the number of nights spent in hospital [17]. At the third interim analysis (after a median follow-up of 5.1 years per patient), the trial was stopped for efficacy. These outcomes were maintained across subgroups in EAST-AFNET 4, including asymptomatic versus symptomatic patients [19], patients with preserved (≥50 %), midrange (40 %–49 %), and reduced (<40 %) LVEF [20], and patients with multiple CV comorbidities [21].

Results from an RWE study of patients with newly diagnosed AF, which included 109,739 patients from the US OptumLabs database and 11,625 patients from the UK Biobank database, also support the use of early rhythm control in patients with AF and high comorbid burden (congestive HF, hypertension, age, diabetes, prior stroke, transient ischemic attack, or thromboembolism, vascular disease, age, sex category [CHA_2_DS_2_-VASc] score ≥ 4) [22]. In this study, patients were classified into several groups: early rhythm control and CHA_2_DS_2_-VASc score ≥ 4; early rhythm control and CHA_2_DS_2_-VASc score 2–3; no early rhythm control and CHA_2_DS_2_-VASc score ≥ 4; or no early rhythm control and CHA_2_DS_2_-VASc score 2–3 [22]. The primary outcome was a composite of all-cause mortality, stroke, or hospitalization with a diagnosis of HF or myocardial infarction (MI). The primary safety outcome was a composite of death, stroke, and serious AEs related to early rhythm control [22]. Most patients received AAD therapy for early rhythm control (range, 86.3 %–94.9 % vs 5.1 %–13.7 % for CA). Most patients received amiodarone (range, 40.1 %–64.8 %), followed by flecainide (range, 6.8 %–16.3 %), sotalol (range, 11.6 %–12.7 %), dronedarone (range, 6.7 %–8.3 %), propafenone (range, 6.8 %–6.9 %), and dofetilide (range, 1.2 %–1.7 %). The results demonstrated a significant reduction in the primary composite outcome in patients with a high comorbidity burden who received early rhythm control compared with those who received no early rhythm control; this reduction was observed in both the US and UK databases, with hazard ratios (HRs) of 0.83 (95 % confidence interval [CI], 0.72–0.95) and 0.77 (95 % CI, 0.63–0.94), respectively. A similar trend was observed in patients with a lower comorbidity burden; however, the reductions were not significant [22]. There was no increase in risk for the primary safety outcome with early rhythm control [22].

RWE data confirming the benefit of early rhythm control in younger patients have been observed in a retrospective population-based cohort study that included 31,220 individuals with AF from the Korean National Health Insurance Service database [16]. Participants received either rhythm or rate control therapy, initiated within 1 year of AF diagnosis. In patients aged <75 years, early rhythm control, compared with rate control, was associated with a lower risk of the primary composite outcome (CV death, ischemic stroke, hospitalization for HF, or MI; HR, 0.80 [95 % CI, 0.72–0.88]) [16]. However, the protective effect of early rhythm control was attenuated with increasing age [16].

Based on the evidence from these recent studies, the 2023 ACC/AHA/ACCP/HRS guidelines assign a class 2a recommendation for early rhythm control in patients with a recent diagnosis of AF (<1 year) to reduce hospitalization, stroke, and mortality [5]. The 2024 ESC guidelines also give a class IIa recommendation to the consideration of a rhythm control strategy within 12 months of diagnosis in selected patients with AF at risk of thromboembolic events to reduce the risk of CV death or hospitalization [6].

### 2.3 Historical perspective of dronedarone

Dronedarone is a multichannel blocker with characteristics of all 4 Vaughan-Williams AAD classes. It blocks sodium (class I), potassium (class III), and calcium (class IV) ion channels and inhibits beta-adrenergic receptors (class II) [23]. The efficacy of dronedarone has been well established in RCTs [24,25]. The phase 3 ATHENA and EURIDIS-ADONIS trials demonstrated that dronedarone significantly reduces CV-related hospitalization or death in patients with AF and delays AF/atrial flutter (AFL), respectively [24,25]. In addition, in the HESTIA trial, which was conducted in patients with permanent pacemakers (*N* = 94), dronedarone was associated with a 59 % relative reduction in AF burden compared with placebo [26]. The safety profile of dronedarone is also well characterized; compared with other AADs, dronedarone carries a reduced risk of proarrhythmic events [11,[27], [28], [29], [30]]. The most commonly reported AEs across 5 placebo-controlled studies of dronedarone were gastrointestinal AEs, with diarrhea experienced by 9 % of participants receiving dronedarone, compared with 6 % receiving placebo [9]. Discontinuation due to gastrointestinal AEs was observed in 3.2 % of participants receiving dronedarone compared with 1.8 % of those receiving placebo [9]. This is, in part, why dronedarone is administered twice daily at 400 mg instead of 800 mg once daily. Dronedarone should also be administered with a substantial meal; however, this is not due to the possibility of gastrointestinal AEs but rather to ensure maximal drug absorption [31]. Based on the 2023 ACC/AHA/ACCP/HRS guidelines, electrocardiogram (ECG) and liver function testing should be conducted when initiating dronedarone, with liver function being retested within 6 months of initiation [5]. Two reported cases of liver injury requiring transplant after dronedarone treatment were reported in 2011, resulting in the European label being updated to recommend frequent liver function testing for people receiving dronedarone. However, data suggest that the rate of liver injury associated with dronedarone is comparable to that of other AADs [11]. Dronedarone is associated with several important drug–drug interactions, including those metabolized by cytochrome P450 enzyme 3A4 (CYP3A4) and P-glycoprotein, and as such, the use of dronedarone is contraindicated with erythromycin and strong CYP3A4 inhibitors. Therefore, it is important to adhere to most recent label recommendations regarding dosing adjustments for concomitant use of dronedarone with treatments that are metabolized by this pathway [9]. A comprehensive review of liver safety and drug–drug interactions associated with dronedarone has been previously published [11].

### 2.4 Dronedarone contraindications

In the US, the use of dronedarone is contraindicated in patients with permanent AF, in those with symptomatic HF with recent decompensation requiring hospitalization, and in those with NYHA class IV HF [9]. In Europe, the use of dronedarone is contraindicated in patients with left ventricular systolic dysfunction and in patients with current or previous episodes of HF [10]. Due to the risk of increased early mortality associated with worsening HF, the 2023 ACC/AHA/ACCP/HRS guidelines state that dronedarone should not be used for maintenance of SR in individuals with NYHA class III/IV HF, or in those who have had an episode of decompensated HF in the past 4 weeks (class 3: harm) [5]. These contraindications are based on results from the phase 3 PALLAS and ANDROMEDA trials [32,33]. The PALLAS trial recruited patients with permanent AF who were subsequently randomized to receive dronedarone or placebo. However, the trial was prematurely halted after recruitment of 3236 of a planned 10,800 patients due to a 2.29-fold increase in the primary endpoint (composite of stroke, MI, systemic embolism, or CV death) in patients receiving dronedarone [32]. A subanalysis revealed a strong association between concurrent digoxin use and the effect of dronedarone on CV death [34]. The ANDROMEDA trial assessed the effectiveness of dronedarone for the treatment of HF. The trial randomized participants who were hospitalized with symptomatic HF and severe left ventricular systolic dysfunction to receive dronedarone or placebo; AF was not an inclusion criterion, and < 40 % of patients had a history of AF at baseline [33]. At the time of randomization, AF was present in 23.2 % of patients in the dronedarone group and 26.8 % of patients in the placebo group. The trial was halted early due to increased mortality in dronedarone-treated patients [33].

### 2.5 Dronedarone for early rhythm control

**Fig. 1:**
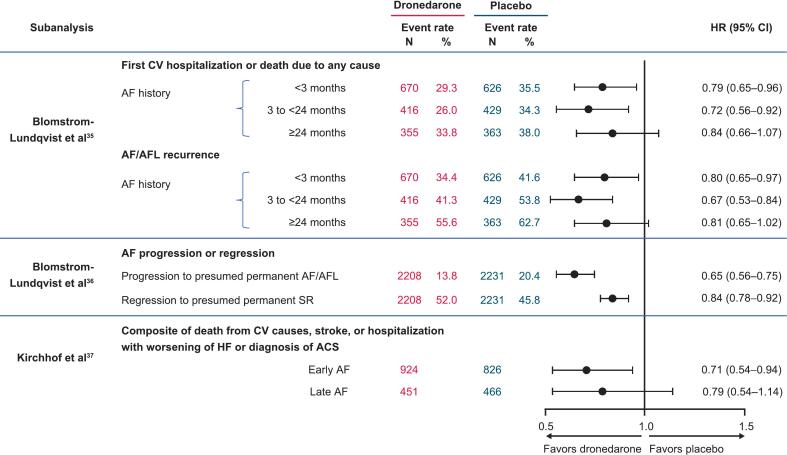
Data supporting the use of dronedarone for early rhythm control [[35], [36], [37]]. ACS, acute coronary syndrome; AF, atrial fibrillation; AFL, atrial flutter; CI, confidence interval; CV, cardiovascular; HF, heart failure; HR, hazard ratio; SR, sinus rhythm.

ATHENA was a randomized, double-blind, placebo-controlled trial in which patients (aged ≥70 years) with paroxysmal or persistent AF or AFL (and additional risk factors for death) were randomized to receive dronedarone 400 mg twice daily or placebo in addition to rate control therapy [24]. Although ATHENA was not a rate versus rhythm control trial [24], several post hoc analyses support the suitability of dronedarone for early rhythm control (Fig. 1) [[35], [36], [37]].

In the first post hoc analysis of the ATHENA trial, patients were categorized as having short, intermediate, or long history of AF/AFL based on the timing of their first known AF/AFL episode before randomization (<3 months, 3–<24 months, or ≥ 24 months, respectively) [35]. The risk of first CV hospitalization or death from any cause (primary endpoint) was lower with dronedarone versus placebo in patients with short and intermediate AF/AFL history (Fig. 1) [35]. In addition, patients with long AF/AFL history had higher rates of AF/AFL recurrence and cardioversion after randomization than did patients with short AF/AFL history [35]. This subanalysis did have some limitations. Although patients were grouped according to timing of their first known AF episode before randomization, the presence of previously unidentified episodes of AF could not be ruled out. In addition, it is unknown what percentages of patients had paroxysmal or persistent AF.

A second post hoc analysis of ATHENA used available ECGs to assess estimated AF/AFL burden over time, cumulative incidence of presumed permanent AF/AFL (AF progression), and presumed permanent SR (AF regression) [36]. To be classified as having presumed permanent AF/AFL, patients were required to have a period of ≥6 months before study end in which all available ECGs showed AF/AFL. To be classified as having presumed permanent SR, patients were required to have a period of ≥6 months before study end in which all available ECGs showed SR [36]. AF/AFL burden was estimated using the modified Rosendaal method to calculate percentage of time in AF/AFL assessed by available ECGs [36]. Compared with those receiving placebo, patients receiving dronedarone had a lower cumulative incidence of presumed permanent AF/AFL, a higher cumulative incidence of presumed permanent SR, and a lower estimated AF/AFL burden over time (Fig. 1) [36].

The most recent post hoc analysis of ATHENA was conducted in patients who met the EAST-AFNET 4 inclusion criteria to evaluate the effectiveness and safety of dronedarone for early rhythm control [37]. Patients were grouped by time of known AF onset (early [≤12 months] vs late [>12 months]). In patients with early AF, compared with placebo, dronedarone was associated with significantly fewer deaths from CV causes, stroke, or hospitalization with worsening of HF or acute coronary syndrome (primary composite endpoint). A similar trend was observed for the primary endpoint in the late AF group, although the results were not significant (Fig. 1). Based on ECG data of patients with early AF, 69.2 % (639/924) of those treated with dronedarone were in SR at 12 months versus 60.8 % (539/886) of those treated with placebo. Dronedarone-treated patients in SR at 12 months were less likely than placebo-treated patients to experience the primary composite endpoint [37]. Additionally, there was a trend toward fewer nights spent in hospital (for any cause and for CV causes) in patients receiving dronedarone versus placebo. This trend was observed in both the early and late AF groups.

To evaluate the use of dronedarone for early rhythm control of AF, the CHANGE AFIB study was designed to determine whether treatment with dronedarone, in addition to usual care, is superior to usual care alone for the prevention of CV hospitalization or death from any cause in patients with first-detected AF [38]. The trial aimed to recruit 3000 patients with first-detected AF. However, enrollment for the study was low, and as of the study completion date (June 30, 2024), only 339 patients were enrolled.

These data provide further support for the use of early rhythm control as indicated in the 2023 ACC/AHA/ACCP/HRS and 2024 ESC guidelines [5,6], and suggest that dronedarone is a suitable agent to initiate early rhythm control in patients with AF.

### 2.6 AAD selection for reducing AF burden and maintaining SR

#### 2.6.1 Recommendations for the use of sotalol

A Cochrane database meta-analysis has established the efficacy of sotalol in maintaining SR after cardioversion of AF [30]. However, in the same report, a meta-analysis of 5 RCTs (*n* = 1882) indicated that sotalol is associated with an increase in all-cause mortality compared with placebo or no treatment (risk ratio [RR], 2.23 [95 % CI, 1.03–4.81]). In addition, a meta-analysis of 12 RCTs (*n* = 2989) found that sotalol is associated with an increased risk of proarrhythmia, including severe symptomatic bradycardia and atrioventricular blocks, compared with placebo or no treatment (RR, 3.55 [95 % CI, 2.16–5.83) [30].

Based on these findings, sotalol has been assigned a class 2b recommendation in the 2023 ACC/AHA/ACCP/HRS guidelines [5]. This is lower than the class 2a recommendation given to other AADs and is a downgrade from the previous class 1 recommendation. The 2023 ACC/AHA/ACCP/HRS guidelines state that for patients with AF without significant baseline QT interval prolongation, hypokalemia, hypomagnesemia, or bradycardia, use of sotalol may be considered for long-term maintenance of SR, with proper dose selection based on kidney function (at initiation) and close monitoring of QT interval, heart rate, serum potassium and magnesium concentrations, and kidney function after initiation [5]. The 2024 ESC guidelines assign sotalol a class IIb recommendation for use as long-term rhythm control in patients with normal LVEF or coronary artery disease to prevent recurrence and progression of AF. However, the guidelines state that close monitoring of QT interval, serum potassium levels, renal function, and other proarrhythmia risk factors is needed [6].

#### 2.6.2 Comparison of efficacy and safety for dronedarone and sotalol

Both dronedarone and sotalol have class II and class III properties. Dronedarone and sotalol both have indications in similar patient populations including those with little or no heart disease, and those with HFpEF or HFmrEF [5,23]. Notably, the use of flecainide and propafenone is only recommended in patients with little or no heart disease, and the use of amiodarone and dofetilide is recommended in patients with varying degrees of HF. In recent years, multiple studies have compared the efficacy and safety of dronedarone and sotalol. Few studies exist that compare dronedarone with other AADs. Therefore, it is appropriate to evaluate the efficacy and safety of dronedarone vs sotalol in detail to enable clinicians to make an informed decision on their use. However, the final decision on which AAD to use should be made on an individual patient basis, considering comorbidities and the safety profile of each AAD. For a comprehensive guide on the use of AADs, we recommend the recently published Practical Compendium of Antiarrhythmic Drugs: A Clinical Consensus Statement of the European Heart Rhythm Association of the ESC [39].

**Fig. 2:**
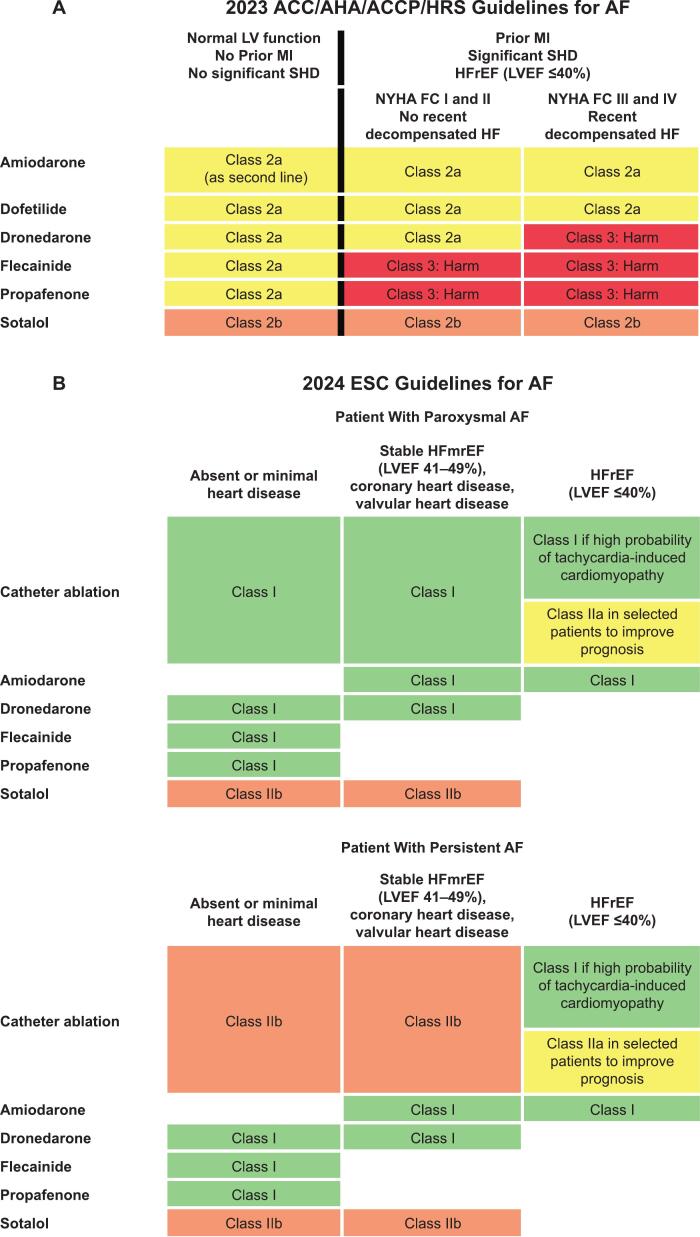
Drug therapy for maintenance of sinus rhythm according to the 2023 ACC/AHA/ACCP/HRS guidelines for AF (A) and options for first time initiation of rhythm control therapy according to the 2024 ESC guidelines for AF^a^ (B). ^a^Final therapy choice should be based on shared decision-making. ACC, American College of Cardiology; ACCP, American College of Clinical Pharmacy; AF, atrial fibrillation; AHA, American Heart Association; ESC, European Society of Cardiology; HF, heart failure; HFmrEF, heart failure with mildly reduced ejection fraction; HFrEF, heart failure with reduced ejection fraction; HRS, Heart Rhythm Society; LV, left ventricular; LVEF, left ventricular ejection fraction; MI, myocardial infarction; NYHA FC, New York Heart Association Functional Class; SHD, significant heart disease.

Based on safety, the class of recommendation for dronedarone as a suitable AAD to reduce AF burden and to maintain SR in AF is higher than that of sotalol (2023 ACC/AHA/ACCP/HRS and 2024 ESC guidelines treatment algorithm; Fig. 2) [5]. Despite having a similar indication, no direct head-to-head RCTs of these agents have been performed; however, several RWE studies have compared dronedarone and sotalol in populations with AF. A retrospective cohort study using data from the US Veterans Health Administration electronic health records (VA EHR) compared effectiveness and safety outcomes between dronedarone and sotalol in propensity score–matched (PSM), AAD-naive patients with AF (*n* = 3106 for both groups; Table 1) [40]. While the risk for CV hospitalization, death, and conductive disorders was similar between the 2 AADs, dronedarone was associated with a lower risk of ventricular proarrhythmia (HR, 0.53 [95 % CI, 0.38–0.74]), bradycardia leading to pacemaker insertion (0.56 [0.36–0.89]), and symptomatic bradycardia (0.56 [0.37–0.87]) [40]. The data from this study were included in a wider prespecified meta-analysis of 4 retrospective observational cohort studies in 4 databases (Optum, MarketScan, VA EHR, Swedish National Patient Register [NPR]) that assessed the comparative safety of dronedarone versus sotalol for the treatment of AF in AAD-naive patients (Table 1) [41]. The primary endpoints were rates of CV hospitalization and ventricular arrhythmia; secondary endpoints included all-cause mortality (VA EHR and Swedish NPR databases only) and bradyarrhythmia associated with syncope/pacemaker implant. Only descriptive statistics were provided for secondary endpoints. The results demonstrated that rates of CV hospitalization (pooled HR [pHR], 0.91 [95 % CI, 0.85–0.97]) and ventricular arrhythmia (pHR, 0.77 [0.69–0.85]) were statistically lower with dronedarone versus sotalol (Table 1). In addition, there was a trend toward reduced mortality (pHR, 0.85 [95 % CI, 0.66–1.10]) and bradyarrhythmia associated with syncope/pacemaker implant (0.71 [0.62–0.80]) with dronedarone versus sotalol. A composite endpoint of all-cause mortality and ventricular arrhythmias assessed in the VA EHR and Swedish NPR showed consistency with both individual endpoints (pHR, 0.72 [95 % CI, 0.60–0.86]).

**Table 1:** Real-world studies and meta-analyses investigating the efficacy and safety of dronedarone versus sotalol.

Study	No. of patients	AF population	Database	Main findings
Pundi et al. [40]	3106 dronedarone-treated patients PSM 1:1 with 3106 sotalol-treated patients	AAD-naive patients with AF	US Veterans Health Administration	With dronedarone vs sotalol • Lower risk of ventricular proarrhythmia • Lower risk of bradycardia leading to pacemaker insertion • Lower risk of symptomatic bradycardia
Singh et al. [41]	23,275 dronedarone-treated patients PSM 1:1 with 23,275 sotalol-treated patients	AAD-naive patients with AF	Optum, MarketScan, Veterans Health Administration Electronic Health Records, Swedish National Patient Register (includes data from Pundi et al.^40^)	With dronedarone vs sotalol • Superiority of dronedarone for rates of CV hospitalization • Superiority of dronedarone for rates of ventricular arrhythmia • Lower rates of bradyarrhythmia associated with syncope/pacemaker implant
Wharton et al. [58]	2224 dronedarone-treated patients PSM 1:1 with 2224 sotalol-treated patients	Patients with AF receiving dronedarone or sotalol after ablation	Merative™	With dronedarone vs sotalol • Lower rates of CV hospitalization at 3, 6, and 12 months • Lower rates of ATA-related hospitalization • Lower rates of hospitalization due to HF • Lower rates of combined proarrhythmia • Lower rates of bradycardic proarrhythmia and need for pacemaker implant
Zeitler et al. [59]	1600 dronedarone-treated patients PSM 1:1 with 1600 sotalol-treated patients Additional subanalysis of 1115 male and 460 female dronedarone-treated patients PSM 1:1 with 1115 male and 460 female sotalol-treated patients	Patients with AF receiving dronedarone or sotalol after ablation	Merative™	With dronedarone vs sotalol • Lower cumulative incidence of all-cause, CV-related, and ATA-/AF-related hospitalizations and pacemaker implants • Lower prevalence rates for all-cause, CV-related, and ATA-/AF-related hospitalizations; all-cause and CV-related ED visits; pacemaker implants; and repeat ablation With dronedarone vs sotalol analyzed by sex • Lower HCRU in both male and female patients post ablation • Lower cumulative incidence of all-cause and CV-related hospitalizations in both male and female patients post ablation • Lower cumulative incidence of ATA-/AF-related hospitalizations in males but not females

AAD, antiarrhythmic drug; AF, atrial fibrillation; ATA, atrial tachyarrhythmia; CV, cardiovascular; ED, emergency department; HCRU, healthcare resource utilization; PSM, propensity score matched.

Although in both RWE studies of dronedarone versus sotalol discussed above [40,41] patients who received a combined total daily sotalol dose <160 mg/d were excluded, the median sotalol dose at the time of event was 160 mg/d across outcomes. These findings suggest that the lowest recommended therapeutic dose of sotalol still carried a significantly increased risk of adverse class II (bradyarrhythmia associated with syncope/pacemaker implant) and class III (ventricular arrhythmia or ventricular proarrhythmia) events compared with dronedarone [40]. This may reflect the increased risk for torsade de pointes and other potentially lethal arrhythmias with sotalol compared with dronedarone, which can result from the effect of class III AADs (such as sotalol and dofetilide) on potassium channels [23,30]. Another contributing factor to the reduced risk of ventricular arrhythmia or ventricular proarrhythmia with dronedarone versus sotalol may be dronedarone's class IV effect on slow-release calcium channels, which may reduce known triggers of ventricular arrhythmia [42,43].

Together, these data support the use of dronedarone over sotalol for treatment of AF based on safety as supported by the 2023 ACC/AHA/ACCP/HRS and 2024 ESC guidelines [5,6].

### 2.7 Use of AADs post ablation

#### 2.7.1 The role of CA as a treatment for AF

In the 2014 AHA/ACC/HRS guidelines, CA was recommended as a class 1 strategy for rhythm control only in patients with symptomatic paroxysmal AF refractory or intolerant to ≥1 class I or class III AAD [7]. However, recent trials show that CA is a more effective treatment than AAD therapy for persistent or paroxysmal AF [18,44,45]. Additionally, early implementation of ablation as a rhythm control strategy is an important factor for improving AF ablation success rates [18,44,[46], [47], [48]]. Based on this evidence, the 2023 ACC/AHA/ACCP/HRS guidelines assign a class 1 recommendation for the use of CA in selected patients (namely, those who are younger with few comorbidities) with symptomatic paroxysmal AF [5]. The 2024 ESC guidelines endorse the use of CA in patients with paroxysmal AF who require rhythm control therapy; a class I recommendation is given to both CA and AAD therapy (with drug choice depending on the presence of HF and comorbidities), based on shared decision-making with the patient (Fig. 2) [6]. In individuals with persistent AF, CA has a class IIb recommendation (Fig. 2). The European Heart Rhythm Association/HRS/Asia Pacific HRS/Latin American HRS recently released a 2024 consensus statement for catheter and surgical ablation of AF [49]. In this statement, CA is rated as beneficial as first-line therapy for patients with symptomatic paroxysmal AF and in those with either paroxysmal or persistent AF who are resistant or intolerant to AADs. The statement further suggests that CA may be a reasonable approach in selected patients, for example, those with asymptomatic AF (paroxysmal or persistent), or as first-line therapy for symptomatic patients with persistent AF [49].

However, clinical trial data revealed that after a first CA procedure, ∼10 % of patients experience recurrence of AF [50]; other data from a US population revealed that 11 % of patients require repeat ablation within 1 year [51]. Evidence from several studies supports the short-term use of AADs versus no AAD use following AF ablation, with results demonstrating a significantly lower risk of early recurrence of AF after ablation with AAD use [[52], [53], [54], [55], [56]]; however, this risk reduction does not predictably lead to a reduction in the risk of recurrence of late atrial arrhythmia [52]. The 2023 ACC/AHA/ACCP/HRS guidelines reflect this, assigning a class 2a recommendation for the use of short-term (3–6 months) AAD therapy after CA in selected patients to reduce early recurrence of atrial arrhythmia and subsequent hospitalization [5]. In addition, the guidelines give a class 1 recommendation that in patients with recurrent symptomatic AF after CA, repeat CA or AAD therapy is useful to improve symptoms and freedom from AF. Results from the ARRC-AF study revealed that after their first CA procedure, 43.6 % of patients received AAD therapy, of whom, 15.4 % received amiodarone, 11 % flecainide, 8.1 % sotalol, 6.9 % dronedarone, 5.7 % dofetilide, and 3.2 % propafenone [57].

#### 2.7.2 Dronedarone after CA

A post hoc subanalysis of the ATHENA trial assessed the efficacy and safety of dronedarone compared with placebo for treatment of paroxysmal/persistent AF/AFL in 196 patients (dronedarone *n* = 90, placebo *n* = 106) who had CA for AF/AFL before study entry [56]. The risk of AF/AFL recurrence was reduced with dronedarone versus placebo (HR, 0.65 [95 % CI, 0.42–1.00]) and the median time to first AF/AFL recurrence was longer (561 vs 180 days). Dronedarone had no effect on time to first CV hospitalization/all-cause death versus placebo (HR, 0.98; 95 % CI, 0.62—1.53).

In an analysis of retrospective PSM post-CA cohorts from Merative™ MarketScan Research Databases, 2224 patients receiving dronedarone were matched 1:1 to patients receiving sotalol [58]. Lower rates of CV hospitalization were observed in patients treated with dronedarone versus sotalol at 3 months (adjusted HR [aHR], 0.73 [95 % CI, 0.61–0.87]), 6 months (0.73 [0.62–0.85]), and 12 months (0.79 [0.69–0.90]) (Table 1) [58]. The lower rates of CV hospitalization in dronedarone- versus sotalol-treated patients were attributable to lower rates of atrial tachyarrhythmia (ATA)–related hospitalization and hospitalization due to HF [58]. Additionally, tolerability was improved in patients treated with dronedarone compared with sotalol, with lower rates of combined proarrhythmia, predominantly driven by lower rates of bradycardic proarrhythmia and need for pacemaker implant (Table 1) [58].

The real-world impact of the use of dronedarone versus sotalol following CA on healthcare resource utilization (HCRU) in patients with AF has been investigated in a retrospective, observational cohort study using Merative™ MarketScan data [59]. After 12 months of follow-up, compared with sotalol, dronedarone was associated with lower cumulative incidence of all-cause, CV-related, and ATA-/AF-related hospitalizations, and pacemaker implants (all *P* < 0.05; Table 1). In addition, the prevalence rates of all-cause, CV-related, and ATA-/AF-related hospitalizations; all-cause and CV-related emergency department visits; pacemaker implants; and repeat ablation were consistently lower with dronedarone versus sotalol (Table 1) [59]. No significant difference was observed in the cumulative incidence rate of repeat CA with dronedarone versus sotalol. A separate analysis further explored these outcomes by assessing differences in HCRU over 12 months according to sex in patients receiving dronedarone versus sotalol following CA (Table 1) [59]. Lower HCRU and lower cumulative incidence of all-cause and CV-related hospitalizations were observed in both male and female populations who received dronedarone versus sotalol. A lower cumulative incidence of ATA-/AF-related hospitalizations was observed in males, but not females, receiving dronedarone [59].

Taken together, when used as rhythm control therapy after CA, dronedarone, compared with sotalol, is associated with reduced rates of AF recurrence; fewer events of all-cause, CV-, and ATA-/AF-related hospitalizations; fewer events of all-cause and CV-related emergency department visits; and fewer pacemaker implants for up to 12 months after ablation.

### 2.8 Ease of use of dronedarone versus other AADs for maintenance of SR

The safety of dronedarone has been extensively studied [27,28]. A retrospective cohort study using PSM populations compared outcomes in people with AF who were treated with dronedarone or other AADs (amiodarone, dofetilide, flecainide, propafenone, or sotalol; ∼50 % used amiodarone) [60]. Dronedarone was associated with significantly lower risk of the primary outcome of CV hospitalization within 24 months after the first drug fill (HR, 0.87 [95 % CI, 0.79–0.96]) and the secondary composite outcome of CV hospitalization or all-cause mortality during follow-up (0.86 [0.78–0.95]) [60]. In an international observational cohort study in patients with AF receiving AAD therapy (patients with NYHA class IV HF were excluded), the effectiveness and safety of dronedarone were compared with that of other AADs commonly used for preventing AF recurrence [61]. In the cohort receiving other AADs, 46.5 % received amiodarone, 42.1 % received flecainide or propafenone, 10.8 % received sotalol, and 0.6 % received other drugs (ajmaline or quinidine). There was no significant difference between dronedarone and other AADs in the risk of first confirmed AF recurrence during the 18-month follow-up period (primary endpoint; aHR, 1.16 [95 % CI, 0.87–1.55]) [61]. Additionally, no significant differences in safety were found between dronedarone and other AADs. Higher rates of mild-to-moderate liver injuries were observed during dronedarone use versus other AADs, although overall, the frequency of AEs and serious AEs was lower with dronedarone.

There is no requirement for dronedarone to be initiated in the inpatient setting. The pharmacokinetic properties of dronedarone allow the same dose (400 mg twice daily, taken orally) to be used in all patients without the need for a loading dose, making administration simple [62]. However, it is important to note that to maximize absorption and subsequent bioavailability, dronedarone should be taken with a substantial meal (as opposed to a light snack) [31].

### 2.9 Consistency of efficacy and safety of dronedarone in different patient populations

Several recent studies have demonstrated that the efficacy and safety profile of dronedarone is consistent across different patient populations. A post hoc analysis of the EURIDIS-ADONIS trials assessed patient characteristics as predictors of dronedarone trough concentrations and characterized the relationship of trough concentrations of dronedarone with its efficacy and safety [63]. Age ≥ 75 years, female sex, lower weight, higher pacemaker use, and higher oral anticoagulant use were associated with above-median trough concentrations [63]. Dronedarone was associated with a significantly lower risk of AF/AFL recurrence compared with placebo in both the below- and above-median trough concentration groups (HR, 0.71 [95 % CI, 0.56–0.91] and 0.63 [0.50–0.81], respectively). No difference in risk of AF/AFL recurrence between the below- and above-median trough concentration groups was observed, and the safety and tolerability of dronedarone were similar between groups [63]. A post hoc analysis of the ATHENA trial assessed the efficacy and safety of dronedarone by age and sex. Compared with placebo, dronedarone significantly reduced the risk of CV hospitalization or death due to any cause and AF/AFL recurrence among patients aged 65–74 years and ≥ 75 years, in both male and female subgroups (Table 2) [64]. Safety was similar regardless of sex, with numbers of reported treatment-emergent AEs (TEAEs) increasing with age [64].

**Table 2:** Post hoc analyses of dronedarone randomized controlled trials in different patient populations.

Post hoc analysis	Parent trial	Population assessment	Main conclusions
Thind et al. [63]	EURIDIS-ADONIS	Patients with above- or below-median dronedarone trough concentrations	Above-median trough concentration was associated with age ≥ 75 years, female sex, lower weight, higher pacemaker use, and higher oral anticoagulant use Risk of adjudicated first AF/AFL recurrence was significantly lower with dronedarone vs placebo in below-median and above-median trough concentration groups No difference in risk of AF/AFL recurrence between the below- and above-median trough concentration groups was observed
Curtis et al. [64]	ATHENA	Age and sex	Dronedarone significantly reduced risk of CV hospitalization or death and AF/AFL recurrence vs placebo among patients aged 65–74 years and ≥ 75 years, regardless of sex
Ma et al. [65]	ATHENA	Asian vs non-Asian patients	Efficacy of dronedarone was consistent in Asian and non-Asian populations Occurrence of TEAEs was higher in Asian population vs non-Asian population, however, comparable safety profiles were observed with dronedarone and placebo in Asian and non-Asian patients
Thind et al. [68]	EURIDIS-ADONIS	Stratified according to renal function	Dronedarone may provide an effective therapeutic option with an acceptable safety profile for people with AF/AFL with renal function ≥45 mL/min, without the need for dose adjustment or continuous monitoring of renal function
Vamos et al. [67]	ATHENA	Stratified according to renal function	Dronedarone reduced risk of first CV hospitalization or death from any cause in individuals with AF/AFL and additional risk factors across a wide range of renal functioning
Handelsman et al. [69]	EURIDIS-ADONIS ATHENA	With and without diabetes	Dronedarone reduced incidence of CV hospitalization/death and AF/AFL recurrence and decreased time to these events in patients with AF/AFL with and without diabetes

AF, atrial fibrillation; AFL, atrial flutter; CV, cardiovascular; TEAE, treatment-emergent adverse event.

Another post hoc analysis of the ATHENA trial compared the efficacy and safety of dronedarone with placebo in Asian versus non-Asian patients (Table 2) [65]. Time to first CV hospitalization or death from any cause was significantly lower for dronedarone versus placebo in both Asian (HR, 0.541 [95 % CI, 0.320–0.914]) and non-Asian (0.768 [0.696–0.848]) populations [65]. Time to AF/AFL recurrence was shorter in the Asian population than the non-Asian population. However, the median time to first AF/AFL event was longer for dronedarone-treated versus placebo-treated patients in both the Asian and non-Asian populations [65]. The overall incidence of TEAEs was higher in the Asian population versus the non-Asian population; however, the incidence of TEAEs was similar between dronedarone and placebo within each population [65]. Another RWE study in an Asian population (National Health Insurance database, Taiwan) compared multiple CV outcomes in people with AF receiving AADs with concomitant non–vitamin K antagonist oral anticoagulants (NOACs) [66]. The study compared outcomes in cohorts of (1) dronedarone versus non-dronedarone AADs, (2) dronedarone versus amiodarone, and (3) dronedarone versus propafenone. Overall, dronedarone was associated with a reduction in stroke, CV death, major adverse CV events, and all-cause mortality when compared with non-dronedarone AADs or amiodarone in this Asian population. There were no differences between dronedarone and propafenone for primary and secondary outcomes [66].

Separate post hoc analyses of the ATHENA [67] and EURIDIS-ADONIS trials [68] assessed dronedarone versus placebo in patients with AF stratified according to renal function. In the ATHENA trial subanalysis, dronedarone significantly lowered the risk of first hospitalization or death from any cause in all patient subgroups with an estimated glomerular filtration rate (eGFR) ≥45 mL/min (as calculated by the Chronic Kidney Disease Epidemiology Collaboration [CKD-EPI] equation), with efficacy similar to that observed in the overall ATHENA primary analysis [67]. This was mirrored in the post hoc analysis of the EURIDIS-ADONIS trials, with a significant reduction in the risk of AF/AFL recurrence observed with dronedarone versus placebo in all eGFR categories ≥45 mL/min (per CKD-EPI) [68]. In both subanalyses, dronedarone was associated with a trend for reduction in the above outcomes versus placebo in patients with eGFR <45 mL/min; however, the reduction was not significant.

Diabetes has been shown to increase the risk of AF. A post hoc analysis of the ATHENA and EURIDIS-ADONIS trials was conducted to assess the effects of dronedarone on outcomes in patients with AF/AFL with or without diabetes [69]. A higher rate of CV hospitalization and death and a shorter median time to the first AF/AFL recurrence were observed in patients with versus without diabetes [69]. However, dronedarone was associated with reduced CV hospitalization rates, delayed time to first CV hospitalization and death, and reduced AF/AFL recurrence versus placebo, both in patients with and without diabetes [69].

Taken together, the evidence presented suggests that an oral dosing regimen of dronedarone 400 mg twice daily, without the need for loading, is effective and well tolerated across different patient populations irrespective of sex, age (>65–74 or ≥ 75 years), ethnicity (Asian or non-Asian), and the presence or absence of comorbidities (impaired renal function; diabetes) while requiring minimal monitoring and no need for initial inpatient administration.

### 2.10 Use of dronedarone and other therapies in patients with HF

AF and HF can often coexist. Having both conditions is associated with worse outcomes than having either one alone [70]. AF can induce, sustain, and worsen HF and the reverse is also true [71]. It is also possible that guideline-directed medical therapy (GDMT) for HF can interact with optimal rhythm control for AF [72]. These interactions can be complicated. For example, GDMT can reduce the incidence of AF; nevertheless, components of GDMT, such as beta-blockers, may only be associated with reductions in rates of CV hospitalization or mortality in those patients who have achieved SR, as compared with use in patients with AF [72]. Notably, GDMT has changed over the last several years. Sacubitril/valsartan and sodium-glucose cotransporter 2 inhibitors are now routinely used; however, HF studies for dronedarone and other therapies, including CA, have not included large numbers of patients receiving these therapies. The 2023 ACC/AHA/ACCP/HRS guidelines assign a class 1 recommendation that arrhythmia-induced cardiomyopathy should be suspected in patients who newly present with HFrEF and AF and an early and aggressive approach to AF rhythm control should be adopted [5].

It is important to recognize the growing use of CA for the treatment of AF in patients with HF, which is in line with clinical trial results demonstrating that CA may result in better outcomes than AAD therapy in some patients with severe HF, for example, HFrEF [73]. In the CASTLE-AF trial, CA was compared with rate or rhythm control therapy in patients with coexisting AF and medically managed NYHA class II, III, or IV HF and LVEF ≤35 % who had an implanted defibrillator [74]. The results showed that CA was associated with a significantly lower rate of the composite endpoint of death from any cause or hospitalization for worsening HF (HR, 0.62 [95 % CI, 0.43–0.87]) [74]. CABANA was an RCT that compared CA with rate or rhythm control therapy for the treatment of AF in patients aged ≥65 years or those aged <65 years who had ≥1 risk factor for stroke [45]. In a subanalysis of patients with NYHA class II or greater HF (778 of 2204 patients enrolled in the original trial), CA resulted in a 36 % relative reduction in the primary composite endpoint of death, disabling stroke, serious bleeding, or cardiac arrest (HR, 0.64 [95 % CI, 0.41–0.99]) and a 43 % relative reduction in all-cause mortality (0.57 [0.33–0.96]) compared with drug therapy alone over 48.5 months of follow-up. Additionally, CA reduced AF recurrence (HR, 0.56 [95 % CI, 0.42–0.74]) [75].

To determine the generalizability of the CABANA trial in patients with AF, a retrospective cohort analysis using OptumLabs Data Warehouse [45] was conducted to assess outcomes in patients in routine practice who would have met trial eligibility. It also examined the association between CA and clinical outcomes in different patient groups stratified according to potential trial eligibility (met eligibility, did not meet inclusion criteria, met an exclusion criterion) [76]. The findings suggested that, compared with rate or rhythm drug therapy, CA was associated with reduction in the primary composite endpoint of all-cause mortality, stroke, major bleeding, or cardiac arrest (HR, 0.75 [95 % CI, 0.70–0.81]), with this finding being particularly prominent in patients who would have been eligible for the trial (0.70 [0.63–0.77]) [76].

The 2023 ACC/AHA/ACCP/HRS guidelines give a class 1 recommendation to CA for appropriate patients with AF and HFrEF receiving GDMT, when there is reasonable expectation of procedural benefit to improve symptoms, quality of life (QoL), ventricular function, and CV outcomes [5]. The guidelines also assign CA a class 2a recommendation when there is again reasonable expectation of benefit to improve symptoms and QoL in appropriate patients with symptomatic AF and HFpEF [5]. In the 2024 ESC guidelines, CA has a class I recommendation in patients with AF and HFrEF with high probability of tachycardia-induced cardiomyopathy to reverse left ventricular dysfunction [6]. The 2024 ESC guidelines also assign CA a class IIa recommendation in selected patients with AF and HFrEF to reduce HF hospitalization and prolong survival [6]. However, some patients, particularly those who are less likely to benefit from CA or those who decide against a procedural approach, may prefer pharmacologic therapy over ablation. In these situations, it is important to differentiate between HFrEF and HFpEF and to ensure that the most appropriate AAD is selected, based on discussion with the patient.

As previously mentioned, in the 2023 ACC/AHA/ACCP/HRS guidelines, dronedarone has a class 2a recommendation for use in patients with AF without recent decompensated HF or severe LV dysfunction for long-term maintenance of sinus rhythm [5]. In patients with NYHA class III or IV HF, or those who have had an episode of decompensated HF in the past 4 weeks, dronedarone has a class 3: harm recommendation [5]. The guidelines also give a class 2a recommendation for the use of amiodarone and dofetilide, and a class 2b recommendation for sotalol in this population. The 2024 ESC guidelines assign dronedarone a class I recommendation to prevent recurrence and progression of AF in patients with AF requiring long-term rhythm control, including those with HFmrEF, HFpEF, ischemic heart disease, or valvular disease [6]; sotalol has the same recommendation but at a class IIb level. The guidelines also recommend that amiodarone be reserved for patients with HFrEF or HFmrEF (class I).

A recent post hoc analysis evaluated different patient groups from the ATHENA trial: (1) those with symptomatic HFpEF or HFmrEF (LVEF >40 %; evidence of structural heart disease; NYHA class II/III, or diuretic use), (2) those with HFrEF or LVEF (≤40 %), and (3) those without HF [77]. Dronedarone was associated with reduced CV events in patients with paroxysmal or persistent AF/AFL and HF when compared with placebo. The results were maintained across the spectrum of LVEF (HFpEF and HFmrEF) [77]. For the primary endpoint of first occurrence of all-cause mortality or CV hospitalization, compared with placebo, dronedarone was associated with reduced risk of death or CV hospitalization (HR, 0.76 [95 % CI, 0.69–0.84]) without heterogeneity based on HF status (*P*_interaction_ > 0.10) [77].

Given the favorable safety profile of dronedarone compared with amiodarone and sotalol and the ease of use of dronedarone, it is reasonable to consider dronedarone as a first-line therapy in patients with HFmrEF, HFpEF, in the absence of NYHA class III/IV or HF with recent decompensation who indicate a preference for AAD therapy over CA. This recommendation aligns closely with the 2024 ESC guidelines, which specifically address treatment of individuals with HFmrEF and HFpEF, whereas in comparison, the 2023 ACC/AHA/ACCP/HRS guidelines recommend AAD therapy for patients with LVEF >40 % who do not present with NYHA class III/IV or who have had recent decompensation.

## 3 Conclusions

The 2023 ACC/AHA/ACCP/HRS and 2024 ESC guidelines provide major updates for the use of rhythm control therapy as a treatment for AF. The 2023 ACC/AHA/ACCP/HRS guidelines were the first to recommend early rhythm control of AF, highlighting the importance of this approach in preventing AF progression, and are now also followed by similar recommendations in the ESC guidelines. The 2023 ACC/AHA/ACCP/HRS guidelines suggest that CA is superior to AAD therapy for first-line rhythm control but recognize the benefit of short-term use of AADs after ablation to reduce the risk of AF recurrence, whereas the ESC guidelines give equal footing for AAD therapy and CA for rhythm control of symptomatic paroxysmal AF based on a shared decision with the patient. Recent data showing increased mortality risk for sotalol have resulted in a downgrade to a class 2b recommendation for this AAD in the 2023 ACC/AHA/ACCP/HRS guidelines (compared with class 2a for all other AADs within their appropriate patient populations), and the 2024 ESC guidelines maintain their lower class IIb recommendation for the use of sotalol compared with AADs with similar indications.

Recent RWE studies and post hoc analyses of key dronedarone RCTs have strengthened the evidence for the safety and efficacy profile of dronedarone as a treatment for patients with HFmrEF, HFpEF, and HFrEF (<40 %) in the absence of NYHA class III/IV or HF with recent decompensation. Data suggest that within these populations, dronedarone is a suitable approach for early-rhythm control of AF, as first-line therapy of AF, and for short term use after CA to reduce the risk of AF recurrence. However, the final choice of AAD should be based on the individual characteristics of the patient, considering comorbidities, AAD safety profiles, and personal choice.

## CRediT authorship contribution statement

**Gerald V. Naccarelli:** Conceptualization, Writing – original draft, Writing – review & editing. **Justin Rackley:** Conceptualization, Writing – original draft, Writing – review & editing. **Giuseppe Boriani:** Conceptualization, Writing – original draft, Writing – review & editing.

## Ethical statement and consent to participate

N/A

## Funding

Medical writing support for this manuscript was funded by 10.13039/100004339Sanofi.

## Declaration of competing interest

Gerald V. Naccarelli is a consultant to Acesion.

Justin Rackley has nothing to disclose.

Giuseppe Boriani reports speaker fees from Bayer, Boehringer Ingelheim, Boston, Daiichi Sankyo, Janssen, and Sanofi outside of the submitted work. He is Principal Investigator of the ARISTOTELES project (Applying ARtificial Intelligence to define clinical trajectorieS for personalized predicTiOn and early deTEction of comorbidity and muLtimorbidity pattErnS), which received funding from the European Union within the Horizon 2020 research and innovation program (Grant No. 101080189).

## Appendix A Supplementary data

Supplementary material Image 1

## References

[1] Colilla S., Crow A., Petkun W., Singer D.E., Simon T., Liu X. (2013). Estimates of current and future incidence and prevalence of atrial fibrillation in the U.S. adult population. Am. J. Cardiol..

[2] Lippi G., Sanchis-Gomar F., Cervellin G. (2021). Global epidemiology of atrial fibrillation: an increasing epidemic and public health challenge. Int. J. Stroke.

[3] Batul S.A., Gopinathannair R. (2017). Atrial fibrillation in heart failure: a therapeutic challenge of our times. Korean. Circ. J..

[4] Tsao C.W., Aday A.W., Almarzooq Z.I., Alonso A., Beaton A.Z., Bittencourt M.S. (2022). Heart disease and stroke statistics-2022 update: a report from the American Heart Association. Circulation.

[5] Joglar J.A., Chung M.K., Armbruster A.L., Benjamin E.J., Chyou J.Y., Cronin E.M. (2024). 2023 ACC/AHA/ACCP/HRS Guideline for the Diagnosis and Management of Atrial Fibrillation: A Report of the American College of Cardiology/American Heart Association Joint Committee on Clinical Practice Guidelines. Circulation.

[6] Van Gelder I.C., Rienstra M., Bunting K.V., Casado-Arroyo R., Caso V., Crijns H. (2024). 2024 ESC guidelines for the management of atrial fibrillation developed in collaboration with the European Association for Cardio-Thoracic Surgery (EACTS). Eur. Heart J..

[7] January C.T., Wann L.S., Alpert J.S., Calkins H., Cigarroa J.E., Cleveland J.C. (2014). 2014 AHA/ACC/HRS guideline for the management of patients with atrial fibrillation: executive summary: a report of the American College of Cardiology/American Heart Association Task Force on practice guidelines and the Heart Rhythm Society. Circulation.

[8] Hindricks G., Potpara T., Dagres N., Arbelo E., Bax J.J., Blomstrom-Lundqvist C. (2021). 2020 ESC guidelines for the diagnosis and management of atrial fibrillation developed in collaboration with the European Association for Cardio-Thoracic Surgery (EACTS): the Task Force for the diagnosis and management of atrial fibrillation of the European Society of Cardiology (ESC). Developed with the special contribution of the European Heart Rhythm Association (EHRA) of the ESC. Eur. Heart J..

[9] Multaq (2025). Prescribing information. sanofi-aventis US LLC. https://products.sanofi.us/multaq/multaq.pdf.

[10] Multaq 400mg film-coated tablets (2025). Summary of Product Characteristics. sanofi. https://www.medicines.org.uk/emc/product/497/smpc/print.

[11] Boriani G., Blomstrom-Lundqvist C., Hohnloser S.H., Bergfeldt L., Botto G.L., Capucci A. (2019). Safety and efficacy of dronedarone from clinical trials to real-world evidence: implications for its use in atrial fibrillation. Europace.

[12] de Vos C.B., Pisters R., Nieuwlaat R., Prins M.H., Tieleman R.G., Coelen R.J. (2010). Progression from paroxysmal to persistent atrial fibrillation clinical correlates and prognosis. J. Am. Coll. Cardiol..

[13] Padfield G.J., Steinberg C., Swampillai J., Qian H., Connolly S.J., Dorian P. (2017). Progression of paroxysmal to persistent atrial fibrillation: 10-year follow-up in the Canadian Registry of Atrial Fibrillation. Heart Rhythm..

[14] Camm A.J., Naccarelli G.V., Mittal S., Crijns H., Hohnloser S.H., Ma C.S. (2022). The increasing role of rhythm control in patients with atrial fibrillation: JACC state-of-the-art review. J. Am. Coll. Cardiol..

[15] Kim D., Yang P.S., You S.C., Jang E., Yu H.T., Kim T.H. (2021). Comparative effectiveness of early rhythm control versus rate control for cardiovascular outcomes in patients with atrial fibrillation. J. Am. Heart Assoc..

[16] Kim D., Yang P.S., You S.C., Jang E., Yu H.T., Kim T.H. (2022). Age and outcomes of early rhythm control in patients with atrial fibrillation: nationwide cohort study. JACC. Clin. Electrophysiol..

[17] Kirchhof P., Camm A.J., Goette A., Brandes A., Eckardt L., Elvan A. (2020). Early rhythm-control therapy in patients with atrial fibrillation. N. Engl. J. Med..

[18] Kuck K.H., Lebedev D.S., Mikhaylov E.N., Romanov A., Geller L., Kalejs O. (2021). Catheter ablation or medical therapy to delay progression of atrial fibrillation: the randomized controlled atrial fibrillation progression trial (ATTEST). Europace.

[19] Willems S., Borof K., Brandes A., Breithardt G., Camm A.J., Crijns H. (2022). Systematic, early rhythm control strategy for atrial fibrillation in patients with or without symptoms: the EAST-AFNET 4 trial. Eur. Heart J..

[20] Rillig A., Magnussen C., Ozga A.K., Suling A., Brandes A., Breithardt G. (2021). Early rhythm control therapy in patients with atrial fibrillation and heart failure. Circulation.

[21] Rillig A., Borof K., Breithardt G., Camm A.J., Crijns H., Goette A. (2022). Early rhythm control in patients with atrial fibrillation and high comorbidity burden. Circulation.

[22] Dickow J., Kany S., Roth Cardoso V., Ellinor P.T., Gkoutos G.V., Van Houten H.K. (2023). Outcomes of early rhythm control therapy in patients with atrial fibrillation and a high comorbidity burden in large real-world cohorts. Circ. Arrhythm. Electrophysiol..

[23] Heijman J., Hohnloser S.H., Camm A.J. (2021). Antiarrhythmic drugs for atrial fibrillation: lessons from the past and opportunities for the future. Europace.

[24] Hohnloser S.H., Crijns H.J., van Eickels M., Gaudin C., Page R.L., Torp-Pedersen C. (2009). Effect of dronedarone on cardiovascular events in atrial fibrillation. N. Engl. J. Med..

[25] Singh B.N., Connolly S.J., Crijns H.J., Roy D., Kowey P.R., Capucci A. (2007). Dronedarone for maintenance of sinus rhythm in atrial fibrillation or flutter. N. Engl. J. Med..

[26] Ezekowitz M.D., Ellenbogen K.A., DiMarco J.P., Kaszala K., Boddy A., Geba G.P. (2015). A placebo-controlled, double-blind, randomized, multicenter study to assess the effects of dronedarone 400 mg twice daily for 12 weeks on atrial fibrillation burden in subjects with permanent pacemakers. J. Interv. Card. Electrophysiol..

[27] Friberg L. (2014). Safety of dronedarone in routine clinical care. J. Am. Coll. Cardiol..

[28] Friberg L. (2018). Ventricular arrhythmia and death among atrial fibrillation patients using anti-arrhythmic drugs. Am. Heart J..

[29] Freemantle N., Lafuente-Lafuente C., Mitchell S., Eckert L., Reynolds M. (2011). Mixed treatment comparison of dronedarone, amiodarone, sotalol, flecainide, and propafenone, for the management of atrial fibrillation. Europace.

[30] Valembois L., Audureau E., Takeda A., Jarzebowski W., Belmin J., Lafuente-Lafuente C. (2019). Antiarrhythmics for maintaining sinus rhythm after cardioversion of atrial fibrillation. Cochrane Database Syst. Rev..

[31] Naccarelli G.V., McKindley D.S., Rashkin J., Ollier C., Reiffel J.A. (2024). Bioavailability of dronedarone tablets administered with or without food in healthy participants. Am. Heart. J. Plus..

[32] Connolly S.J., Camm A.J., Halperin J.L., Joyner C., Alings M., Amerena J. (2011). Dronedarone in high-risk permanent atrial fibrillation. N. Engl. J. Med..

[33] Kober L., Torp-Pedersen C., McMurray J.J., Gotzsche O., Levy S., Crijns H. (2008). Increased mortality after dronedarone therapy for severe heart failure. N. Engl. J. Med..

[34] Hohnloser S.H., Halperin J.L., Camm A.J., Gao P., Radzik D., Connolly S.J. (2014). Interaction between digoxin and dronedarone in the PALLAS trial. Circ. Arrhythm. Electrophysiol..

[35] Blomstrom-Lundqvist C., Marrouche N., Connolly S., Corp Dit Genti V., Wieloch M., Koren A. (2020). Efficacy and safety of dronedarone by atrial fibrillation history duration: insights from the ATHENA study. Clin. Cardiol..

[36] Blomstrom-Lundqvist C., Naccarelli G.V., McKindley D.S., Bigot G., Wieloch M., Hohnloser S.H. (2023). Effect of dronedarone vs. placebo on atrial fibrillation progression: a post hoc analysis from ATHENA trial. Europace.

[37] Kirchhof P., Camm A.J., Crijns H.J.G.M., Piccini J.P., Torp-Pedersen C., McKindley D.S. (2025). Dronedarone provides effective early rhythm control: post-hoc analysis of the ATHENA trial using EAST-AFNET 4 criteria. Europace.

[38] Pokorney S.D., Nemeth H., Chiswell K., Albert C., Allyn N., Blanco R. (2025). Design and rationale of a pragmatic randomized clinical trial of early dronedarone versus usual care to change and improve outcomes in persons with first-detected atrial fibrillation - the CHANGE AFIB study. Am. Heart J..

[39] Merino J.L., Tamargo J., Blomström-Lundqvist C., Boriani G., Crijns H.J.G.M., Dobrev D. (2025). Practical compendium of antiarrhythmic drugs: a clinical consensus statement of the European Heart Rhythm Association of the European Society of Cardiology. Europace.

[40] Pundi K., Fan J., Kabadi S., Din N., Blomstrom-Lundqvist C., Camm A.J. (2023). Dronedarone versus sotalol in antiarrhythmic drug-naive veterans with atrial fibrillation. Circ. Arrhythm. Electrophysiol..

[41] Singh J.P., Wieloch W., Reynolds S.L., Blomstrom-Lundqvist C., Sandhu A.T., Camm A.J. (2023). The Heart Rhythm 2023 Annual Meeting.

[42] Moro S., Ferreiro M., Celestino D., Medei E., Elizari M.V., Sicouri S. (2007). In vitro effects of acute amiodarone and dronedarone on epicardial, endocardial, and M cells of the canine ventricle. J. Cardiovasc. Pharmacol. Ther..

[43] Varró A., Takács J., Németh M., Hála O., Virág L., Iost N. (2001). Electrophysiological effects of dronedarone (SR 33589), a noniodinated amiodarone derivative in the canine heart: comparison with amiodarone. Br. J. Pharmacol..

[44] Andrade J.G., Wells G.A., Deyell M.W., Bennett M., Essebag V., Champagne J. (2021). Cryoablation or drug therapy for initial treatment of atrial fibrillation. N. Engl. J. Med..

[45] Packer D.L., Mark D.B., Robb R.A., Monahan K.H., Bahnson T.D., Poole J.E. (2019). Effect of catheter ablation vs antiarrhythmic drug therapy on mortality, stroke, bleeding, and cardiac arrest among patients with atrial fibrillation: the CABANA randomized clinical trial. JAMA.

[46] Friedman D.J., Field M.E., Rahman M., Goldstein L., Sha Q., Sidharth M. (2021). Catheter ablation and healthcare utilization and cost among patients with paroxysmal versus persistent atrial fibrillation. Heart. Rhythm. O2..

[47] Monahan K.H., Bunch T.J., Mark D.B., Poole J.E., Bahnson T.D., Al-Khalidi H.R. (2022). Influence of atrial fibrillation type on outcomes of ablation vs. drug therapy: results from CABANA. Europace.

[48] Wazni O.M., Dandamudi G., Sood N., Hoyt R., Tyler J., Durrani S. (2021). Cryoballoon ablation as initial therapy for atrial fibrillation. N. Engl. J. Med..

[49] Tzeis S., Gerstenfeld E.P., Kalman J., Saad E.B., Sepehri Shamloo A., Andrade J.G. (2024). 2024 European Heart Rhythm Association/Heart Rhythm Society/Asia Pacific Heart Rhythm Society/Latin American Heart Rhythm Society expert consensus statement on catheter and surgical ablation of atrial fibrillation. Europace.

[50] Kuck K.H., Furnkranz A., Chun K.R., Metzner A., Ouyang F., Schluter M. (2016). Cryoballoon or radiofrequency ablation for symptomatic paroxysmal atrial fibrillation: reintervention, rehospitalization, and quality-of-life outcomes in the FIRE AND ICE trial. Eur. Heart J..

[51] Piccini J.P., Sinner M.F., Greiner M.A., Hammill B.G., Fontes J.D., Daubert J.P. (2012). Outcomes of Medicare beneficiaries undergoing catheter ablation for atrial fibrillation. Circulation.

[52] Chen W., Liu H., Ling Z., Xu Y., Fan J., Du H. (2016). Efficacy of short-term antiarrhythmic drugs use after catheter ablation of atrial fibrillation-a systematic review with meta-analyses and trial sequential analyses of randomized controlled trials. PLoS One.

[53] Duytschaever M., Demolder A., Phlips T., Sarkozy A., El Haddad M., Taghji P. (2018). PulmOnary vein isolation With vs. without continued antiarrhythmic Drug trEatment in subjects with Recurrent Atrial Fibrillation (POWDER AF): results from a multicentre randomized trial. Eur. Heart J..

[54] Leong-Sit P., Roux J.F., Zado E., Callans D.J., Garcia F., Lin D. (2011). Antiarrhythmics after ablation of atrial fibrillation (5A Study): six-month follow-up study. Circ. Arrhythm. Electrophysiol..

[55] Pokushalov E., Romanov A., Artyomenko S., Baranova V., Losik D., Bairamova S. (2013). Cryoballoon versus radiofrequency for pulmonary vein re-isolation after a failed initial ablation procedure in patients with paroxysmal atrial fibrillation. J. Cardiovasc. Electrophysiol..

[56] Vamos M., Calkins H., Kowey P.R., Torp-Pedersen C.T., Corp Dit Genti V., Wieloch M. (2020). Efficacy and safety of dronedarone in patients with a prior ablation for atrial fibrillation/flutter: insights from the ATHENA study. Clin. Cardiol..

[57] Saksena S., Ken-Opurum J., McKindley D.S., Preblick R., Rashkin J., Aldaas O.M. (2025). Arrhythmia recurrence and rhythm control strategies after catheter ablation of newly diagnosed atrial fibrillation (ARRC-AF study). JACC. Clin. Electrophysiol..

[58] Wharton J.M., Piccini J.P., Koren A., Huse S., Ronk C.J. (2022). Comparative safety and effectiveness of sotalol versus dronedarone after catheter ablation for atrial fibrillation. J. Am. Heart Assoc..

[59] Zeitler E.P., Stein D., Preblick R., Kabadi S.M., McKindley D.S., Rashkin J. (2025). Health care resource utilization with dronedarone versus sotalol following catheter ablation in adults with atrial fibrillation. Clin. Cardiol..

[60] Goehring E.L., Bohn R.L., Pezzullo J., Tave A.K., Jones J.K., Bozzi S. (2020). Outcomes associated with dronedarone use in patients with atrial fibrillation. Am. J. Cardiol..

[61] Khachatryan A., Merino J.L., de Abajo F.J., Botto G.L., Kirchhof P., Breithardt G. (2022). International cohort study on the effectiveness of dronedarone and other antiarrhythmic drugs for atrial fibrillation in real-world practice (EFFECT-AF). Europace.

[62] Dorian P. (2010). Clinical pharmacology of dronedarone: implications for the therapy of atrial fibrillation. J. Cardiovasc. Pharmacol. Ther..

[63] Thind M., McKindley D.S., Reiffel J.A., Naccarelli G.V., Stewart J., Kowey P.R. (2022). Predictors of dronedarone plasma drug concentrations and effect on atrial fibrillation/atrial flutter recurrence: analyses from the EURIDIS and ADONIS studies. Clin. Cardiol..

[64] Curtis A.B., Zeitler E.P., Malik A., Bogard A., Bhattacharyya N., Stewart J. (2022). Efficacy and safety of dronedarone across age and sex subgroups: a post hoc analysis of the ATHENA study among patients with non-permanent atrial fibrillation/flutter. Europace.

[65] Ma C., Lin J.L., Bai R., Sun Y., Nam G.B., Stewart J. (2022). Effect of dronedarone in the treatment of atrial fibrillation in the Asian population: post hoc analysis of the ATHENA trial. Clin. Ther..

[66] Wu V.C., Wang C.L., Huang Y.C., Tu H.T., Huang Y.T., Huang C.H. (2023). Cardiovascular outcomes in patients with atrial fibrillation concomitantly treated with antiarrhythmic drugs and non-vitamin K antagonist oral anticoagulants. Europace.

[67] Vamos M., Oldgren J., Nam G.B., Lip G.Y.H., Calkins H., Zhu J. (2022). Dronedarone vs. placebo in patients with atrial fibrillation or atrial flutter across a range of renal function: a post hoc analysis of the ATHENA trial. Eur. Heart. J. Cardiovasc. Pharmacother..

[68] Thind M., Zareba W., Atar D., Crijns H., Zhu J., Pak H.N. (2022). Efficacy and safety of dronedarone versus placebo in patients with atrial fibrillation stratified according to renal function: post hoc analyses of the EURIDIS-ADONIS trials. Clin. Cardiol..

[69] Handelsman Y., Bunch T.J., Rodbard H.W., Steinberg B.A., Thind M., Bigot G. (2022). Impact of dronedarone on patients with atrial fibrillation and diabetes: a sub-analysis of the ATHENA and EURIDIS/ADONIS studies. J. Diabetes Complicat..

[70] Wang T.J., Larson M.G., Levy D., Vasan R.S., Leip E.P., Wolf P.A. (2003). Temporal relations of atrial fibrillation and congestive heart failure and their joint influence on mortality: the Framingham Heart Study. Circulation.

[71] Verhaert D.V.M., Brunner-La Rocca H.P., van Veldhuisen D.J., Vernooy K. (2021). The bidirectional interaction between atrial fibrillation and heart failure: consequences for the management of both diseases. Europace.

[72] Newman J.D., O'Meara E., Bohm M., Savarese G., Kelly P.R., Vardeny O. (2024). Implications of atrial fibrillation for guideline-directed therapy in patients with heart failure: JACC state-of-the-art review. J. Am. Coll. Cardiol..

[73] Turagam M.K., Garg J., Whang W., Sartori S., Koruth J.S., Miller M.A. (2019). Catheter ablation of atrial fibrillation in patients with heart failure: a meta-analysis of randomized controlled trials. Ann. Intern. Med..

[74] Marrouche N.F., Brachmann J., Andresen D., Siebels J., Boersma L., Jordaens L. (2018). Catheter ablation for atrial fibrillation with heart failure. N. Engl. J. Med..

[75] Packer D.L., Piccini J.P., Monahan K.H., Al-Khalidi H.R., Silverstein A.P., Noseworthy P.A. (2021). Ablation versus drug therapy for atrial fibrillation in heart failure: results from the CABANA trial. Circulation.

[76] Noseworthy P.A., Gersh B.J., Kent D.M., Piccini J.P., Packer D.L., Shah N.D. (2019). Atrial fibrillation ablation in practice: assessing CABANA generalizability. Eur. Heart J..

[77] Vaduganathan M., Piccini J.P., Camm A.J., Crijns H., Anker S.D., Butler J. (2022). Dronedarone for the treatment of atrial fibrillation with concomitant heart failure with preserved and mildly reduced ejection fraction: a post-hoc analysis of the ATHENA trial. Eur. J. Heart Fail..

