# Supplementary material for: New recommendations for rhythm control—What has changed in the 2023 ACC/AHA/ACCP/HRS and 2024 ESC guidelines for atrial fibrillation, and where does dronedarone fit in?
Source: Am Heart J Plus. 2025 Oct 20;60:100645. doi: 10.1016/j.ahjo.2025.100645 (PMC12811750; doi:10.1016/j.ahjo.2025.100645)
Supplement: Supplementary file 1 — Supplementary material [file mmc1.docx]

**Supplementary Materials**

**Supplementary Methods**

For each recommendation in the 2023 American College of Cardiology (ACC)/American Heart Association (AHA)/American College of Clinical Pharmacy (ACCP)/Heart Rhythm Society (HRS) guidelines, a class of recommendation is assigned based on certainty of benefit in proportion to risk; these are class 1 (strong), class 2a (moderate), class 2b (weak), class 3: no benefit (moderate), and class 3: harm (strong). In addition, the Level of Evidence (LoE) is provided for each recommendation as follows: LoE A (high-quality); LoE B-N (moderate, derived from randomized studies); LoE B-NR (moderate, derived from nonrandomized studies); LoE C-LD (based on limited data); and LoE C-EO (based on clinical opinion). The 2024 European Society of Cardiology (ESC) guidelines also provide the class (strength) of recommendation, using class I (recommended or indicated), class IIa (should be considered), class IIb (may be considered), and class III (not recommended). LoE is defined as A (multiple randomized controlled trials [RCTs] or meta-analysis), B (single RCT or multiple large nonrandomized studies), or C (expert opinion or small studies/retrospective studies or registries).
